# Earlswood as It Is

**Published:** 1890-03-01

**Authors:** 


### Earlswood As It Is.

(By Our Lunacy Commissioner.)

Close to the Brighton line, twenty miles and a-half from town, stands a large building in the Italian palazzo style of architecture, and wayfarers are told that it is the Earlswood Asylum for Idiots. We were quite lately privileged to spend a long day at the asylum, and under the guidance of its talented and courteous medical officers we made a most careful inspection of the wards, schoolrooms, workshops, and grounds. Although we arrived before nine o'clock in the morning, we found both officers on duty, one of them actually in the wards ready for any emergency.

Towards the close of last century it began to be recognised in England that something ought to be done for the lunatics, and early in the present century we commenced the building of our county asylums, a process which has not yet ceased, nor is it likely to cease for some time to come. But it was not until the century was fifty years old that the idiots had anything special done for them. Even yet the provision is totally inadequate, and these unfortunate creatures are stowed away in workhouses or are seen wandering aimlessly about the wards of our county asylums. A friend of the idiots arose in the person of Andrew Reed, D.D., a Nonconformist minister. He pleaded their cause with the eloquence of a Peter the Hermit, and did not forget to devote much money to the work as well as his time and words. The result was the founding of the Earlswood Asylum and the laying of the foundation stone by the late Prince Consort.

The visitor to this fine institution enters a spacious entrance hall, in which are cases containing toys, ornaments, articles of fretwork, baskets, brushes, &c. All these are made by the idiots, and the various things are offered for sale at very low prices, the proceeds being devoted to some fund connected with the asylum, so that anyone unable or unwilling to subscribe can still help on the good work by making purchases. We noticed in the hall also some good specimens of cabinet-making and a grand model of a ship. We learned that not only our own international exhibitions but also the Paris one has had Earlswood productions among the exhibits. Immediately behind the entrance is a fine hall, large enough to seat six hundred people, and used as a chapel and dining-room. The roof of this hall is extremely good, and reminds us of the large hall at Hampton Court. The walls are embellished with the names of many kind benefactors. Passing further we reach the playroom and recreation hall, in which there is a capacious stage and much fine scenery. The kitchen is close by, and the manner in which the meals are served is worthy of much praise.

The main service corridors or galleries run east and west, and out of them open the day rooms and dormitories, all of these being extremely bright and homelike. There is no painful reminder of the "institution" about them. The east and west wings are chiefly kept for private patients, and are really a series of sitting-rooms and bedrooms, fitted up in the most comfortable manner and bright with ornaments and tasteful decoration. We should have liked to see more flowers about; but probably the expense of the extension of the glass-houses is at present an insuperable objection to such an increase. With the girls' wardrobe room we were particularly struck. It is indeed a marvel of neatness and order, and is not the only part of the establishment which speaks well for the watchful eye of the matron, Mrs. Noble.

We spent much of our time in the schools. In many cases the advance made by the children was wonderful. Reading, writing, and arithmetic are carefully taught, and, where any special aptitude is evinced, drawing also. Of course there are different grades in idiocy, as in everything else, and in the worst cases any form of education would be quite hopeless. One room on each side is set apart for the cripples; their wants have been carefully studied; dormitories open straight out of their day-rooms, and their airing courts are at hand.

The Sick Hospital is close to the main building, and is well arranged for the treatment of ordinary diseases, but it could hardly be safely used in the event of an outbreak of fever or small-pox. It is awful to think what havoc such an outbreak might make here, and it is greatly to be desired that some generous individual will come forward and begin the good work of founding a hospital for infectious cases. Surely there must be many in England who would subscribe the necessary two or three thousand pounds for this purpose did they but know what a pressing want it is. It is evident that the medical care of the patients is never lost sight of at Earlswood, and the nursing is good. There are upwards of six hundred patients, or "boys" and "girls," as they are all called, in residence now, and the asylum must afford rare opportunities for the study of infantile diseases. Of course ordinary lunatics are not admitted. Charity cases are elected by the votes of the subscribers, and paying cases are taken in at rates varying from sixty pounds a-year to three hundred, according to the accommodation required, and those paying the higher rates may have their own servants and use of carriage.

A very considerable item in the expenses at Earlswood is the teaching staff; but then on the efficiency of this a great deal depends. Without this provision these waifs and strays of humanity would soon degenerate still further in the scale, and hopelessness take the place of hope. It is evident that in the head schoolmaster and head schoolmistress Earlswood possesses most painstaking and zealous officers.

It is at first glance rather curious that the boys very greatly exceed the girls in number, and more than one far-fetched theory has, from time to time, been invented to account for the preponderance of the boys; but the real, obvious reason, and certainly the chief one, is that boys are everywhere more troublesome than girls, whether idiotic or no; and girls can be kept at home, and can be gradually broken in to domestic work, while boys are nuisances to everyone around, and so are sent to the asylum.

Various trades are vigorously carried on at Earlswood. Sixteen boys work in the carpenter's shop, seventeen or eighteen with the basketmaker, forty-five are in the shoemaking department, nine are with the printer, and twenty-four with the tailor. In many instances the work turned out by the boys is extremely creditable, and shows how carefully the paid staff attend to their duties. The farm buildings are well arranged, and between twenty and thirty of the boys are daily employed in the health-giving, if not pecuniarily profitable, occupation of farming. Forty cows supply the inmates with milk, and about two hundred pigs enjoy a comparatively easy life until the time comes for their being converted by a process of animal chemistry into idiots.

Gymnastic exercise now forms part of every school education, and it is not forgotten at Earlswood, while football and cricket matches are of frequent occurrence. Theatricals, dances, picnics, and band performances are often held, and many of the pupils are allowed to spend summer holidays with their friends.

An institution like Earlswood cannot be stationary, and its onward march in efficiency and reputation under Dr. Jones is one of the most striking facts in connection with its history. The managing committee were lucky in securing such a medical superintendent, and he, in his turn, was equally fortunate in meeting with an assistant medical officer so energetic and kind-hearted as Dr. Cooper.

It is sad to think that the income of this institution cannot be got to keep pace with the claims on it. Possibly, and we hope certainly, this only needs to be widely known to be remedied. We understand that the chairman would gladly receive subscriptions if sent to him to the Office, 36, King William Street, E.C.

